# Molecular characterization, structural analysis and determination of host range of a novel bacteriophage LSB-1

**DOI:** 10.1186/1743-422X-7-255

**Published:** 2010-09-28

**Authors:** Yaming Chai, Hongyan Xiong, Xiangyu Ma, Liqing Cheng, Guorong Huang, Zhonglin Rao Lin Zhang

**Affiliations:** 1 Department of Epidemiology, Faculty of Preventive Medicine, Third Military Medical University, Chongqing 400038, China

## Abstract

**Background:**

Bacteriophages (phages) are widespread in the environment and play a crucial role in the evolution of their bacterial hosts and the emergence of new pathogens.

**Results:**

LSB-1, a reference coliphage strain, was classified as a member of the Podoviridae family with a cystic form (50 ± 5 nm diameter) and short tail (60 ± 5 nm long). The double stranded DNA was about 30 kilobase pairs in length. We identified its host range and determined the gp17 sequences and protein structure using shotgun analysis and bioinformatics technology.

**Conclusions:**

Coliphage LSB-1 possesses a tailspike protein with endosialidase activity which is probably responsible for its specific enteroinvasive *E.coli *host range within the laboratory.

## Background

Bacteriophages (phages) are widespread in the environment and play a crucial role in the evolution of their bacterial hosts and the emergence of new pathogens. They have enormous potential for the development of new drugs, therapies and environmental control technologies, such as natural, non-toxic alternatives for controlling bacterial pathogens. Recent interest in phages has been stimulated by studies that demonstrate the efficacy of phages in preventing and treating infections [1-3].

Phages are readily isolated from water samples in the environment. Some of the isolated phages have shown broad-host range interaction with the bacterial isolates and others have shown either species or strain level specificity. Both polyvalent phages and non-polyvalent phages are morphologically and genetically diverse [4,5]. They are efficient at host recognition but there is no single method of adsorption, and different phages employ different strategies [6,7]. Identification of the mechanisms of adsorption and the host ranges of different phages may allow genetic manipulation to alter the phage host binding profile artificially. To gain a better understanding of the biological properties of phages, we have sequenced the genome of the gp17 from phage LSB-1, which was isolated from sewage samples. We determined its host range and analyzed its 3-dimensional structure to identify possible functional domains.

## Results

### Coliphage morphology

**Figure 1:**
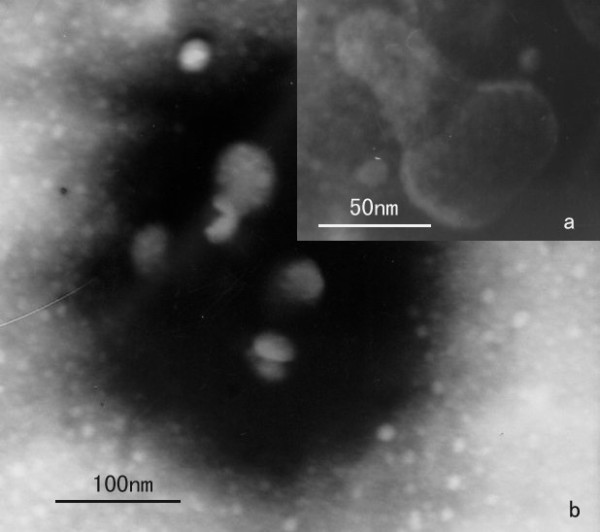
**Electron micrograph of phage LSB-1**. Electron micrographs of the phages with a short, stout tail. The polyhedral nature of the viral head is shown.

The LSB-1 phage has a cystic form of 50 ± 5 nm in diameter, with a short noncontractile tail 60 ± 5 nm long (Fig. 1). It is classified as a member of the Podoviridae family [8-10].

### Nucleic acid characterization

**Figure 2:**
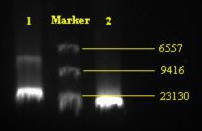
**Agarose gel electrophoresis of DNA of phage LSB-1**. Lane 1: whole genome DNA digested by Nhe I. Lane 2: whole genome DNA without digestion. Molecular size markers (kb) are HindIII-digested lambda DNA.

LSB-1 coliphage nucleic acid was sensitive to Dnase I, but resistant to Rnase A and S1 nuclease (Fig. 2). It was concluded that all the extracts contained double-stranded linear DNA.

### Preliminary overview of the coliphages genome

**Figure 3:**
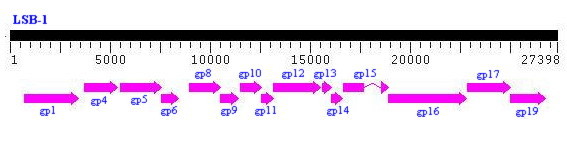
**Comparative genomic arrangements of coliphage LSB-1 with K1F**. Genome of coliphage LSB-1 is aligned. Arrows indicate functions of the ORFs.

**Figure 4:**
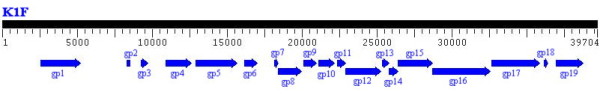
**Comparative genomic arrangements of coliphage LSB-1 with K1F**. Genome of coliphage K1F is aligned. Arrows indicate functions of the ORFs.

The shotgun sequencing and a primer-walking method was used to assemble the whole linear LSB-1 genome of approximately 30 kb. Fifteen major potential ORFs were identified, all of which could be assigned functions based on homology with corresponding genes in the K1F coliphage in a BLAST search (Fig. 3 and 4).

### Genome relationship of LSB-1 and other phages

**Figure 5:**
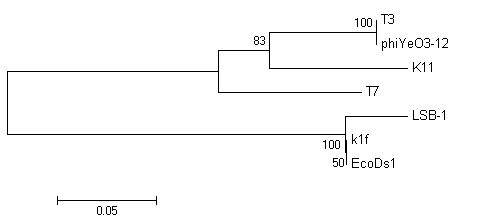
**Phylogenetic analysis of the capsid protein (gp10) from seven phages in the T7 supergroup**. The alignment of whole sequence was used to construct the neighbor-joining tree. The scale bar represents 0.05 fixed mutations per amino acid position. Bootstrap values based on 1000 computer-generated tree are indicated at the nodes.

**Figure 6:**
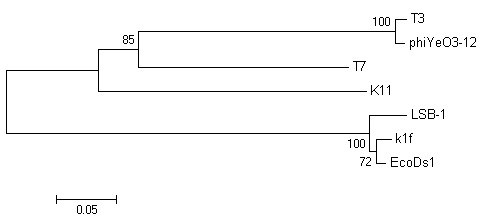
**Phylogenetic analysis of the tail tubular protein (gp12) from seven phages in the T7 supergroup**. The alignment of whole sequence was used to construct the neighbor-joining tree. The scale bar represents 0.05 fixed mutations per amino acid position. Bootstrap values based on 1000 computer-generated tree are indicated at the nodes.

**Figure 7:**
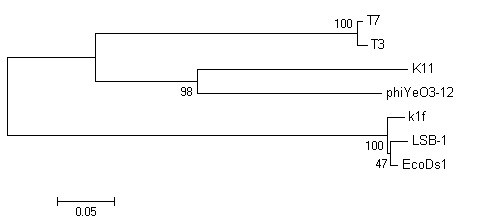
**Phylogenetic analysis of the internal virion protein (gp16) from seven phages in the T7 supergroup**. The alignment of whole sequence was used to construct the neighbor-joining tree. The scale bar represents 0.05 fixed mutations per amino acid position. Bootstrap values based on 1000 computer-generated tree are indicated at the nodes.

**Figure 8:**
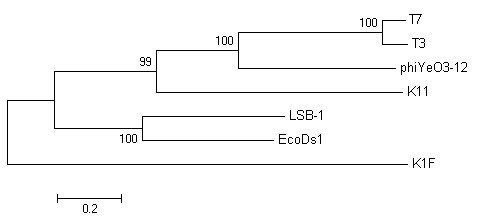
**Phylogenetic analysis of the endosialidase (gp17) from seven phages in the T7 supergroup**. The alignment of whole sequence was used to construct the neighbor-joining tree. The scale bar represents 0.2 fixed mutations per amino acid position. Bootstrap values based on 1000 computer-generated tree are indicated at the nodes.

We chose four important proteins, capsid protein (gp10), tail tubular protein (gp12), internal virion protein D (gp16), and endosialidase (gp17), which are present in phage T7 supergroup members, to build phylogenetic trees. As shown in Fig. 5, 6, 7 and 8, trees built using different proteins are broadly similar. Coliphages LSB-1, EcoDs1, and K1F closely clustered towards the end of the group and phages T7, T3, phiYeO3-12 and K11 cluster within another branch of the same group. These data suggest that LSB-1 is most closely related to the K1F-like phages, and should be classified as a new member of the K1F supergroup.

### Gp17 DNA sequencing and function analysis

**Figure 9:**
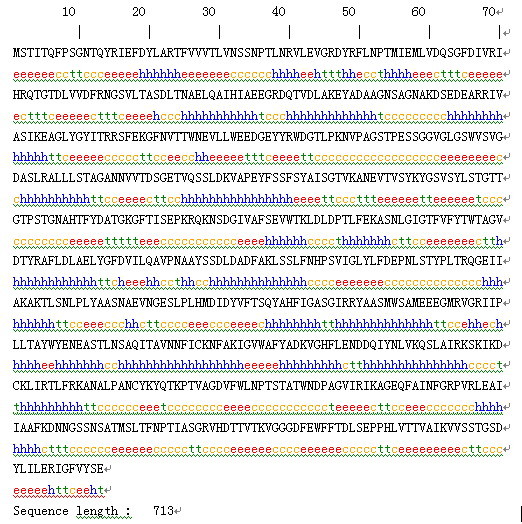
**Secondary structure prediction of gp17 protein**.

**Figure 10:**
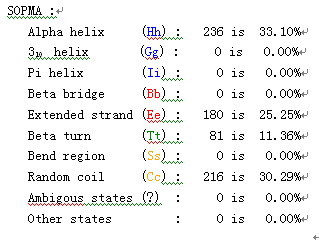
**Supplements and reports of Fig. 9, the secondary structure of gp17 protein comprised 33.1% Alpha helix(Hh), 25.25% Extended strand(Ee) and 30.29% Random coil(Cc)**.

As shown in Fig. 9 and 10, the secondary structure of gp17 protein was composed of 33.10% Alpha helix (Hh), 25.25% Extended strand (Ee) and 30.29% Random coil (Cc).

The PHYRE program generated several possible templates classified as best fit when the gp17 sequence was submitted. All of these templates included the sequences of hydrolase as one of its domains. It also provided the SCOP codes, E-values and estimated precision values for the predicted models. Table 1 shows these parameters provided by different templates.

**Table 1:** Parameters provided by different templates

No	SCOP Code	E-value	Estimated Precision	Classification
1	c2c1lA_	1.1	70%	Hydrolase
2	d7a3ha_	4.2	45%	glycosidases
3	c2cksA_	4.6	40%	Hydrolase
4	c1wkyA_	16	10%	Hydrolase

**Figure 11:**
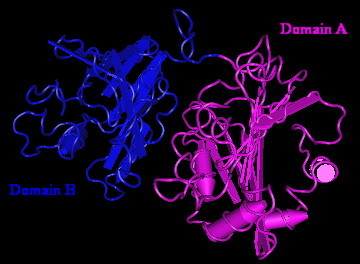
**Computer-generated 3-dimensional modeling of gp17 protein**. Worm algorithm representation of the 3-dimensional model showing the two main domains. The larger Domain A is homologous to hydrolase sequences. The smaller Domain B is homologous to the sequence of the anchoring domain.

The endosialidase 3-dimensional structure was chosen to generate coordinates for the Gp17 of LSB-1 based on an E-value of 1.1 and an estimated precision of 70%. Using the PHYRE program, it was difficult to find another closer coordinate template for Gp17 of LSB-1 even though an E-value of less than 2e-6 was considered [11-13]. Gp17 of LSB-1 included endosialidase amino acid residues from Thr290 to Glu713, with the with the exception of forty-one residues (Ser 465 to Trp475, Tyr497 to Val512, and Lys543 to Leu562). These forty-one residues did not match corresponding sequences within the prediction server. The Gp17 3-dimensional structure model predicted two domains: Domain A with hydrolase activity connected by an intervening unstructured sequence to Domain B. The later domain may have a host-anchoring function. A worm algorithm representation of the model is shown in Fig. 11. Domain A, the larger of the two domains, includes residues Thr290 to Phe528 and corresponds to the N-terminal domain of endosialidase. It comprises 4 α helices and 9 β strands with intermittent unstructured intervening regions. The arrangement is characteristic of crystallized hydrolase. Domain B is composed of 1 α helix and 9 β strands with intermittent unstructured intervening regions. It includes residues Gln542 to Glu713 and corresponds to the C-terminal domain of endosialidase. There were no predicted structural constraints in the connecting region between domains A and B which included the residues of Tyr529 to Asp541.

**Figure 12:**
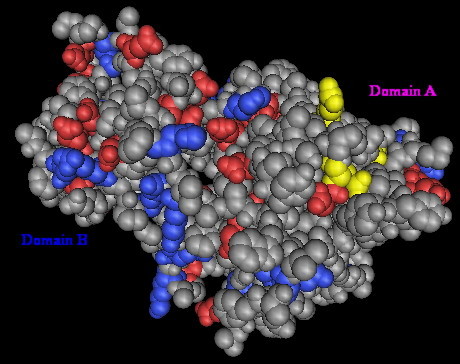
**Computer-generated 3-dimensional modeling of gp17 protein**. Molecular surface presentation of the predicted catalytic pocket of endosialidase. The active site triad (yellow), Glut405, Arg415 and Arg487, is the amidase reaction site of endosialidase.

**Figure 13:**
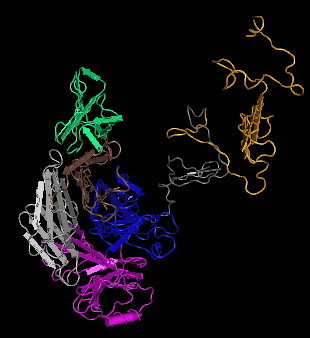
**Worm algorithm representation of the model of endosialidase of K1F**.

**Figure 14:**
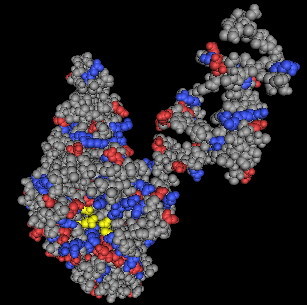
**Molecular surface presentation of K1F endosialidase**. The active site triad (yellow) is Glut581, Arg596 and Arg647.

The molecular surface structure of the Gp17 is presented in Fig. 12. It shows the catalytic pocket in Domain A comprising the catalytic triad, Glut405, Arg415, and Arg487, identical to that found in the endosialidase family of enzymes. Domain A is shown topographically in front of Domain B. The connecting region was difficult to see in this representation because of its opaqueness. Fig. 13 and Fig. 14 show the worm algorithm structures of Gp17and the KIF endosialidase respectively.

## Discussion

Previous studies have suggested that gene 17 from the T7-like and K1F-like phage may be playing an important role in host range recognition processes [14,15], but little work has been done on comparing their molecular characterization. K1F-like phages are known to possess tailspike proteins with endosialidase activity that degrades polySia with high substrate specificity. These tailspike-associated enzymatic activities enable the phages to penetrate the capsular layer and are important determinants of the bacteriophage host range [16]. The T7-like phage encodes a tail fiber protein that specifically recognizes and binds to lipopolysaccharide [17] and recognizes *E. coli *B and many *E. coli *K-12 strains.

It was expected that molecular characterization would provide evidence for a host adsorbing mechanism of coliphage LSB-1. We found that the LSB-1 has some common features of the phage K1F supergroup. A tailspike protein with endosialidase activity is implicated in allowing a specific enteroinvasive *E.coli *host range. Insertion of such an endosialidase gene into a non-polyvalent virulent phage may artificially increase its host range to enteroinvasive *E.coli*. Using manipulation of the phage genome to kill pathogenic bacteria has broad implications for the welfare of both man and animals. Our results may inform new ways of genetic manipulation of phages to alter their host binding profile.

## Materials and methods

### Purification of phage particles

Bacteriophage LSB-1 was propagated in liquid culture. The EIEC strain 8401 was infected with phages at a multiplicity of infection of 0.1. Following complete lysis of the host cells, cell debris was removed by centrifugation at 4000 × *g *for 10 min, and phage particles in the supernatant were concentrated by adding polyethylene glycol 6000 and NaCl to achieve final concentrations of 10% and 1.0 M, respectively. Phage particles were collected by centrifugation at 8000 × *g *for 10 min. Pellets were re-suspended in 0.01× the original volume of sterile SM buffer (5.8 g sodium chloride, 2 g magnesium sulphate, 100 mg gelatin, 50 mL 1 mol·L-1 Tris (pH 7.5) and 945 mL distilled water). For isopycnic centrifugation, the phage suspension was placed on a cesium chloride gradient stepwise using three solutions whose densities were 1.45, 1.50, and 1.70, respectively. After centrifugation for 60 min at 150,000 × *g*, the phage band was withdrawn and dialyzed against 10 mM Tris-HCl (pH 7.5) containing 10 mM MgSO. The purified phage (approximately 10^11^PFU/ml) was stored at 4°C until use.

### Host range determination

Seventeen bacterial strains, listed in Table 2, were tested for sensitivity against the isolated phages. The Spot Test [18] was used to determine the host range of the phage. Lytic activity was examined following overnight incubation at 37°C and recorded on a scale as follows: (N) no plaques, (T) turbid plaques, (C) clear plaques. To obtain an accurate estimate of relative phage lytic activity, all host range determinations were carried out simultaneously using a single high-titer stock of purified bacteriophage and all host cells were incubated at 37°C in LB medium.

**Table 2:** Phage and bacterial strains used in the study and host range spectrum of the bacteriophages

No	Species	Strain	Plaque formation
1	Escherichia	285^a^	N
2	Escherichia	ATCC 25922^b^	N
3	Escherichia	ATCC 8099^b^	N
4	Escherichia	ATCC 10536^b^	N
5	Escherichia	JM109^c^	N
6	Escherichia	BL21^c^	N
7	Escherichia	HDSα^c^	N
8	Escherichia	O157H7 44752^d^	N
9	Escherichia	EIEC 8401^d^	C
10	Escherichia	EIEC ATCC 43893^d^	C
11	Staphylococcus aureus	ATCC 6538^b^	N
12	Staphylococcus aureus	ATCC 26001^b^	N
13	Salmonella typhi	ATCC 50071^b^	N
14	Pseudomonas aeruginosa	ATCC 10145^b^	N
15	Shigella dysenteriae	ATCC 13313^b^	N
16	Α-hemolytic streptococcus	ATCC 32213^b^	N
17	Β-hemolytic streptococcus	ATCC 32210^b^	N

Obtained from ^a^Zhenghui L, Institute of Medical Equipment, Academy of Military Medical Science, China; ^b^Bacterial culture center, Institute of Microbiology and Epidemiology, Academy of Military Medical Science, China; ^c^Jing a. Department of Microbiology, Third Military Medical University, China; ^d^Bacterial culture center, Institute of Epidemiology and Microbiology, Chinese Academy of Preventive Medicine.

C = clear plaque; T = turbid plaque; N = no plaque.

### Electron microscopy

To examine phage LSB-1 morphology, 50 μl of purified viral stock solution was fixed by addition of 50 μl of 0.5% glutaraldehyde in 4% paraformaldehyde and a drop of this solution was placed on a carbon coated copper grid After waiting 30 min for settlement, excess liquid was removed and the grid was allowed to dry. A drop of 2% phosphotungstic acid was added for 2 min before excess was removed with filter paper before drying and then examination by TEM (Hitachi, model S-800) at an acceleration voltage of 45 kV.

### Nucleic acid characterization

The method used was based upon that described by Sambrook and Russell [19]. Bacteriophage from the concentrated solutions were lysed with the addition of EDTA (final concentration 20 mmol·L-1), proteinase K (final concentration 50 μg·mL-1) and SDS (final concentration 0.5%) and incubation at 56°C for 1 h. The nucleic acid was purified using phenol, phenol/chloroform and chloroform extraction. The final aqueous phase was dialyzed overnight against Tris EDTA buffer (TE). In this method, nucleic acid yield was estimated by agarose gel electrophoresis and comparison of ethidium bromide stain intensity with known DNA standards (Hind III cut lambda phage DNA, New England Biolabs Inc, USA).

The nucleic acid extracts were diluted to a standard concentration of ~20 ng·μL-1. Approximately 250ng of each extract was subjected to digestion with DNase I (Sigma Aldrich), RNase A (Sigma Aldrich) and S1 nuclease (Promega). All reactions were terminated with the addition of EDTA (10 mmol·L-1 final concentration) and analyzed using 0.8% agarose gel electrophoresis at 5 V cm-1.

### Gp17 DNA sequencing and analysis

The coliphage genome was sequenced by the shotgun method. Genomic DNA was sheared by sonication, cloned into pUC18 and sequenced with an ABI 3700 automated DNA sequencer, to give 13-fold coverage of the genome. Sequences were assembled into contigs, and gaps linked using a primer-walking technique (Kaczorowski and Szybalski, 1998) [20]. Potential open reading frames (ORFs) were predicted using ORF Finder http://www.ncbi.nlm.nih.gov/projects/gorf/ and manual correction. Translated ORFs were used in a BLAST search against the Swiss-Prot http://us.expasy.org/tools/blast and NCBI protein databases http://blast.ncbi.nlm.nih.gov/Blast.cgi?CMD=Web&PAGE_TYPE=BlastHome. Protein secondary structures were predicted with SOPMA http://npsa-pbil.ibcp.fr/cgi-bin/npsa_automat.pl?page=npsa_sopma.html.

Phylogenetic trees were constructed using Mega 4 software following published protocols [21,22].

The amino acid sequence of endosialidase was determined and compared with the protein structure family databases PDB [23], SCOP [24,25], and PFAM [26] to identify the most suitable template structure. The eventual template structure was taken from PDB. Three-dimensional models were created using PHYRE http://www.sbg.bio.ic.ac.uk/~phyre/ by mapping the coordinates of the template structure with aligned residues of the endosialidase. Computer-generated three-dimensional models were viewed and analyzed using CN3 D 4.1 application software programs obtained from http://www.ncbi.nlm.nih.gov/Structure/CN3D/cn3d.shtml.

## Conclusions

In summary, a typical Podoviridae morphology and the double-stranded nature of its DNA give the coliphage LSB-1 some common features with the phage K1F supergroup. It possesses a tailspike protein with endosialidase activity which is probably responsible for its specific enteroinvasive *E.coli *host range within the laboratory.

## Competing interests

The authors declare that they have no competing interests.

## Authors' contributions

YC and HX conceived of the study, carried out the experimental work, analysis and drafted the manuscript. XM and LC participated in its design and coordination and helped to draft the manuscript. GH and ZR participated in its design and experimental work. LZ participated in coordination of the study. All authors read and approved the final manuscript.
